# Supplementary material for: From sequence to enzyme mechanism using multi-label machine learning
Source: BMC Bioinformatics. 2014 May 19;15:150. doi: 10.1186/1471-2105-15-150 (PMC4229970; doi:10.1186/1471-2105-15-150)
Supplement: Additional file 2 — Java code of ml2db. Additional file ml2db_code.tar.gz contains the Java source code to run the multi-label machine learning experiments and save the results to database. The code’s Javadoc is included. [file 1471-2105-15-150-S2.zip › additional file 2/ml2db/utils/doc/allclasses-frame.html]

All Classes


# All Classes

- AbstractContinousDistribution
- AbstractDistribution
- AllDatabaseUtilsTests
- AllMapUtilsTests
- AllStatsUtilsTests
- AllUtilsTests
- ArrayUtils
- BooleanArrayPool
- ClientHttpRequestUtils
- CollectionUtils
- CollectionUtilsTest
- CommandLineMenu
- CommandOption
- Data
- DbConn
- DbConnectionSingleton
- DbConnPropsTest
- DbCreator
- DbCreatorTest
- DbManaged
- DbManager
- DbManagerSingleton
- DbManagerTest
- DbReader
- DbReaderTest
- DbUtils
- DbUtilsTest
- DbWriter
- DbWriterTest
- Diff
- Difference
- *DoubleFunction*
- EntrezUtils
- FileUtils
- FileUtilsTest
- GuiUtils
- *Initialisable*
- Initialised
- *IntFunction*
- ListUtils
- ListUtilsTest
- LoggerCreator
- LogUtils
- Managed
- Manager
- MapOfMaps
- MapOfMapsTest
- MapUtils
- MapUtilsTest
- MersenneTwister
- NumberUtils
- NumberUtilsTest
- OneToMany
- OneToManyMap
- OneToManyMapTest
- OptionUtils
- Pareto
- ParetoTest
- PathUtils
- PersistentObject
- Pool
- PropertiesUtils
- PseudoTruncatedPareto
- PseudoTruncatedParetoSingleton
- PseudoTruncatedParetoTest
- RandomApp
- RandomElement
- RandomEngine
- RandomJava
- RandomSeedable
- RandomShuffle
- RandomUtilsTest
- Ranecu
- Ranlux
- Ranmar
- ReflectionUtils
- ReflectionUtilsTest
- RegExpUtils
- RegExpUtilsTest
- ResultSetUtils
- Scripts
- Set
- Set
- SetTest
- SetUtils
- SetUtils
- SimpleDOMParser
- SimpleDOMParserTest
- SimpleRadioButtonPanel
- SqlUtils
- SqlUtilsTest
- StatUtils
- StatUtilsTest
- StringPool
- StringUtils
- StringUtilsTest
- SupersetsManager
- SupersetsManager
- SupersetsManagerTest
- SystemUtils
- Table
- TableColumn
- TableCreator
- TableCreatorTest
- TableManager
- TableMap
- TableMapTest
- TableReader
- TableReaderTest
- TableRow
- TableRowTest
- TableTest
- TableWriter
- TableWriterTest
- TimeUtils
- TimeUtilsTest
- TruncatedPareto
- TruncatedParetoTest
- UniformRandomSingleton
- UniformRandomSingletonTest
- UniformRandomUtils
- UniformRandomUtilsTest
- Utils
- UtilsTest
- VectorUtils
- WebUtils
- WebUtilsTest
- XmlNode
- XmlNodeTest
- XmlSearcher
- XmlSearcherTest
- XMLUtils
- XmlUtilsTest
